# Religio Medici

**Published:** 1887-08-27

**Authors:** 


### Religio Medici.

(*Concluded from p.* 345, *vol. ii.*)

Sir Thomas devoutly held the severe doctrine of election, that God out of His mere good pleasure has arbitrarily chosen some to enjoy eternal life, and condemned others to everlasting punishment. He is also sufficiently mediæval to believe that all who had never heard the name of Christ must incur damnation, even those seers and philosophers in whose works he delighted; and the idea grieves his tolerant soul. "It is hard," he ventures to remark, "to place those souls in hell, whose worthy lives do teach us virtue on earth. How strange to them will sound the history of Adam, when they shall suffer for him they never heard of!"

But this is a belief so foreign to his nature that he cannot heartily hold it. Therefore he comforts himself with the assurance that the human mind cannot realise, and so cannot with justice speak of the nature of God.

"I believe," he says, "that many are saved, who to man seem reprobated, and many are reprobated, who, in the opinion and sentence of man, stand elected. There will appear, at the last day, strange and unexpected examples, both of His justice and His mercy; and therefore to define either, is folly in man, and insolency even in the devils. Those acute and subtle spirits in all their sagacity, can hardly divine who shall be saved; which if they could indeed prognosticate, their labours were at an end, nor need they compass the earth, seeking whom they may devour." And again he states his belief that "those who do confine the church of God either to particular churches, nations, or families, have made it far narrower than our Saviour ever meant it."

This uncharitable and presumptuous condemnation is the one thing of which he speaks bitterly. Alluding to the belief of each church and sect that in its tenets alone lie the things necessary to salvation, he quaintly remarks: "There must be therefore more than one St. Peter; particular churches and sects usurp the gates of heaven, and turn the key against each other; and thus we go to heaven against each other's wills, conceits, and opinions, and, with as much uncharity as ignorance, do err, I fear, in point not only of our own, but one another's salvation."

Sir Thomas is altogether of a kindly and charitable nature. It shines through all the first part of his work, which deals with faith; and is exemplified yet more in the second, which is devoted entirely to "the noble virtue of love." In speaking of this he goes from the general sentiment of love for all mankind to the particular affection he feels for friends and kindred. Of the form of the emotion to which the name of love is now specially and peculiarly applied he knows nothing. "I never yet cast a true affection on a woman," he confesses; but in return he claims, "I have loved my friend as I do virtue, my soul, my God." And in a noble passage on friendship, which I cannot forbear quoting at large, he describes this love in words which would satisfy the most exacting nature:—

"There are wonders in true affection. It is a body of enigmas, mysteries, and riddles; wherein two so become one, as they both become two: I love my friend before myself, and yet methinks I do not love him enough. Some few months hence my multiplied affection will make me believe I have not loved him at all. When I am from him, I am dead till I be with him; when I am with him, I am not satisfied, but would still be nearer unto him. United souls are not satisfied with embraces, but desire to be truly each other; which being impossible, their desires are infinite, and must proceed without a possibility of satisfaction. Another misery there is in affection; that whom we truly love like our own selves, we forget their looks, nor can our memory retain the idea of their faces: and it is no wonder, for they are ourselves, and our affection makes their looks our own. This noble affection falls not on vulgar and common constitutions, but on such as are marked for virtue. He that can love his friend with this noble ardour will in a competent degree affect all. Now, if we can bring our affections to look beyond the body, and cast an eye upon the soul, we have found out the true object, not only of friendship, but of charity; and the greatest happiness that we can bequeath the soul is that wherein we all do place our last felicity, salvation; which, though it be not in our power to bestow, it is in our charity and pious invocations to desire, if not procure and further. I cannot contentedly frame a prayer for myself in particular, without a catalogue of my friends; nor request a happiness wherein my sociable disposition doth not desire the fellowship of my neighbour. I never hear the toll of a passing-bell, though in my mirth, without my prayers and best wishes for the departing spirit. I cannot go to cure the body of my patient, but I forget my profession, and call unto God for his soul."

This is the substance of the old physician's creed—love to all mankind, and the desire to help them in body and in soul. He deprecates indeed the idea of making a merit of good works, but instead urges it as a privilege that a Christian should be proud to seize; but beyond all opportunity of bestowing temporal good, he longs for the salvation of every soul with which his own comes in contact. Though he has given up, or thinks he has given up the heresy of Origen, he is still at heart a Universalist; he would not willingly see any condemned to that eternity of misery in which he so devoutly though sadly believed. This is the belief to which the longings of his tender heart guide him: yet he does not hold to it obstinately, perhaps because his soul's insight leads him beyond all merely dogmatic formulas; and he thus concludes his work:—"This is the tenor of my belief; wherein, though there be many things singular, and to the humour of my irregular self, yet, if they square not with maturer judgment, I disclaim them, and do no further favour them than the learned and best judgments shall authorise them."

